# “It’s very hard, but I’ll manage.” Educational aspirations and educational resilience among recently resettled young refugees in Norwegian upper secondary schools

**DOI:** 10.1080/17482631.2020.1785694

**Published:** 2020-12-09

**Authors:** Brit Lynnebakke, Lutine de Wal Pastoor

**Affiliations:** a Section for Trauma, catastrophes and forced migration - adults and elderly, Norwegian Centre for Violence and Traumatic Stress Studies (NKVTS), Oslo, Norway; b Danish Research Centre for Migration, Ethnicity and Health (MESU), Section for Health Services Research, Department of Public Health, University of Copenhagen, Copenhagen K, Denmark

**Keywords:** Young refugees, educational resilience, educational aspirations, motivation, young migrants’ education

## Abstract

**Purpose:** This article explores the question: *what shapes young refugees’ often high educational aspirations*? A sense of mastery and future hope are among the numerous factors that can positively predict school achievements. High aspirations may thus contribute to both young refugees’ improved educational outcomes and wellbeing.

**Method:** We discuss findings from semi-structured interviews in three Norwegian upper secondary schools in light of theories and research into the often high educational aspirations of immigrant youth.

**Results:** Several of the study’s findings support the dual frame of reference theory which has been used in prior discussions of migrants’ aspirations. However, we argue that aspirations can be supported not only by comparisons with the country of origin, but also by comparisons with different stages of the refugee experience.

**Conclusions:** The findings suggest that temporal aspects of high aspirations should be empirically and theoretically explored further. Moreover, this and other studies’ findings on young refugees suggest that research into the educational aspirations of young immigrants should address/control for migration category.

## Introduction

“It’s very hard, but I’ll manage.”

The statement above was made by Dahabo,^1^ a young refugee girl from Somalia who arrived in Norway as an unaccompanied refugee minor after a one year long flight. Four years later, she attends upper secondary school, and is determined to succeed in Norway. Previous research shows that young refugees often demonstrate strong resilience, positive future expectations, and high motivation at school (e.g., Bartlett et al., 2017; Blanchet-Cohen et al., 2017; Oppedal et al., 2017; Pastoor, 2015; Peterson et al., 2017; Shakya et al., 2010). This is despite that young refugees often face complex educational and psychosocial challenges due to little or interrupted education and “the refugee experience” (Pastoor, 2015, 2017). The refugee experience involves various pre-, trans- and post-flight experiences (Stein, 1981).^2^ The overarching research question of the article *is what may shape young refugees’ high educational aspirations*?

### The migration context

Within research on refugees, the term “refugee background” is well established (e.g., Naidoo et al., 2018). In this article, “refugee background people” denotes “persons who have come to Norway for flight reasons, and includes also family reunified immigrants to these” (Aalandslid & Enes, 2012, p. 8, our translation). The research participants had obtained residency in Norway and encompass previous asylum seekers, quota refugees, and individuals who were family reunified to a refugee. The interviewees were recently resettled students, i.e., they had lived 6 years or less in Norway, following the definition of The Norwegian Directorate of Education.

In 2017, when most of the interviews were conducted, 217 200 refugee background people lived in Norway, comprising 4% of Norway’s total population. Most came from Somalia, Iraq, Syria, Eritrea, and Afghanistan. As in many European countries, Norway had a strong increase in asylum seekers in 2015 (Huddleston & Wolffhardt, 2016). After 2015, there has been a sharp decrease in asylum applications in Norway (UDI, 2019).

### The education context

The ten-year compulsory Norwegian schooling comprises primary school (grades 1–7) and lower secondary school (grades 8–10). Upper secondary school provides 3 years of general education, or 4 years of vocational education and training (including 2 years of apprenticeship). Upon finishing lower secondary education, all students have a right to upper secondary education, which nearly all students make use of, including recently arrived language minority students.

Young immigrants have a higher dropout rate in upper secondary education than their Norwegian-born peers (Markussen et al., 2011; Østberg, 2010; Utdanningsdirektoratet, 2016). This has led to new educational provisions for newcomers. Recently arrived students (aged 16‒24) who have completed Norwegian lower secondary school (or its equivalent abroad) but need further training may prepare for mainstream upper secondary education by attending a voluntary one-year *preparatory class* at an upper secondary school. There, they are taught compulsory school subjects, with a special emphasis on learning Norwegian.^3^

All interviewees in this study attended upper secondary school, either in a mainstream class or in a preparatory class.

### The refugee experience and education

Research on young refugees in Norway has especially focused on unaccompanied minors. A recent review of this field (Svendsen et al., 2018, p. 78) calls for more research attention on young refugees and education, not least because of young refugees’ higher drop-out rates. Among the most vulnerable for early school leaving are newcomer students who arrive in Norway late in their teens. A recent Nordic comparative register study (Dunlavy et al., 2020) found that by age 25, young refugees in Scandinavia had substantially lower upper secondary school completion rates (just above 50%) compared to native-born peers (80–90%).

Extensive research from many countries exists on educational outcomes and experiences of young immigrant and descendant youth (“the second generation”). As Ferede (2010) states, “knowledge specific to the resettled refugee experience is often lost within the folds of aggregated educational research” (p. 79). Koehler and Schneider (2019, p. 2) underscore that research on refugees and education can build on and benefit greatly from the vast existing knowledge on descendants and education. However, they point out that the refugee experience may have an impact on education (Cerna, 2019, see also Ferede, 2010; Lynnebakke et al., 2020).

What may these distinct experiences involve? First, many refugee late arrivals have had little or interrupted education because of the flight and political instability and war in the country of origin (Koehler & Schneider, 2019). Furthermore, the developmental tasks of adolescence may become more difficult due to the often traumatic and stressful nature of the refugee experience (Fazel et al., 2012; Pastoor, 2015). Many refugees have had potentially traumatizing experiences before and during flight (Kohli & Mather, 2003; Pastoor, 2015) and experience exile-related stress, which increase vulnerability to psychological distress and post-traumatic stress disorder (PTSD) (Fazel & Stein, 2002). Meanwhile, challenges and future hope can coexist during resettlement (see also Shakya et al., 2010). Concurrent to encountering many challenges, the resettlement period can be the first period in a very long time where young refugees experience greater predictability and personal control over their lives, compared to often prolonged periods of uncertainty before flight, during flight and during the asylum-seeking process. The structure and regularity of school attendance can contribute to a healing process after trauma (Hayward, 2017; Rutter, 2006). Whilst school is important for most young people, school potentially have additional meanings for young refugees. Studies on children underscore that schools potentially contribute to refugee and asylum seeking children’s emotional and social needs, local integration, future hope, and focus on future plans and aspirations (Anderson, 2003; Hek, 2005; Svensson & Eastmond, 2013). Upper secondary school might be a time particularly characterized by a focus on shaping one’s future—being the final school years where one considers one’s future vocation.

## Theoretical framework and previous research

### Educational resilience

We approach our data through the lenses of an ecological model of development (Bronfenbrenner, 1979), and a holistic approach to resilience which entails that psychosocial adjustment and resilience upon resettlement are determined not only by individual traits, but also influenced by relational and environmental factors (Betancourt & Khan, 2008; Masten, 2001; Ungar, 2012). We frame the fact that many young refugees display high motivation, hard work at school, and positive future expectations despite numerous challenges within the concept of *educational resilience*. Educational resilience has been defined as “the heightened likelihood of educational success despite personal vulnerabilities and adversities brought about by environmental conditions and experiences” (Wang et al., 1997, p. 4). Furthermore, educational resilience has been “conceptualized not as the product of a single precipitating event, but of continuous interaction between an individual and characteristic features of the environment” (Wang et al., 1997, p. 4, see also Bahram et al., 2014).

The relationship between educational aspirations and educational achievements is complex (Khattab, 2015, p. 746). However, a sense of mastery and future hope are among the numerous factors that can positively predict school achievements (Bahram et al., 2014; Day et al., 2010; Lewin-Epstein et al., 2014; Suarez-Orozco et al., 2009). Within resilience research, Walsh (2012) underlines that a combination of a positive outlook and a supportive environment enables individuals to deal with life’s challenges. High aspirations and positive future expectations may therefore not “only” contribute to wellbeing, but also to improved educational outcomes.

### Educational aspirations

There is no universal consensus on the right definition of *educational aspirations* (Trebbels, 2015). Trebbels (2015) points out that “A variety of terms like plans, decisions or preferences are commonly used—sometimes interchangeably” (p. 37), whilst other definitions underline hope (e.g., DeMoss, 2013). We conceptualize educational aspirations as comprising both future hope and agency, which drives goal-oriented behaviour to work towards the fruition of these aspirations.

Research on educational aspirations and achievements has found that on a group level, immigrant and descendant youth from many country backgrounds have higher educational aspirations than the native population (e.g., Bakken & Hyggen, 2018; Suarez-Orozco et al., 2009).^4^ For many country backgrounds, high aspirations also coexist with lower educational achievements, compared to the native population (Østberg, 2010; Salikutluk, 2016; Teney et al., 2013). Some country backgrounds also attain higher educational levels than the native population with similar socioeconomic backgrounds (e.g., Tjaden & Hunkler, 2017). These findings have led to several theories that explain high aspiration patterns as related to “the migrant experience” (Østberg, 2010; cf. Salikutluk, 2016; Tjaden & Hunkler, 2017). Many of these studies encompass a variety of country backgrounds or focus on labour migrants with specific country backgrounds. In our discussion of possible reasons for high aspirations, we refer to both the “refugee” experience and “the migration experiences”. Obviously, “labour migrants” and “refugees” are extremely broad categories. Coarsely put, we use “the migration experience” term to refer to the fact that refugee youth and other migrant youth may share similar experiences and conditions, e.g., related to learning a new language, cultural differences, disrupted social bonds and changed family dynamics in the new context (Kao, 1999).

Several theories and explanations have been proposed to explain the often higher educational aspirations of immigrants and their children, and the overrepresentation of certain country backgrounds in higher education compared to natives with similar socioeconomic backgrounds. The explanations are not mutually exclusive but may operate at the same time and/or reinforce each other (Salikutluk, 2016).

First, the *immigrant optimism* theory argues that “immigrants are a positively selected group, as they were willing to leave their home countries with the goal of socio-economic improvement” (Salikutluk, 2016, p. 583). This is especially applicable to labour migrants, but also to some extent to refugees (Østberg, 2010) (cf. Crawley & Skleparis, 2018; Erdal & Oeppen, 2018). The theory emphasizes migration as an intergenerational project for social mobility. Low socioeconomic status in the new country is seen as temporary until mobility is achieved through children’s education and later occupations (Salikutluk, 2016). A second, related, explanation is the *dual frame of reference* theory (Suárez-Orozco & Suárez-Orozco, 1995) which proposes that immigrants compare their lives both to people in their countries of origins and settlement.^5^ The dual frame of reference can support continued high aspirations despite current challenges (Østberg, 2010).

Third, the *blocked opportunities* theory proposes that “structural and social barriers to educational and labour market success spur high educational ambitions as a reaction” (Salikutluk, 2016, p. 583). An expectation of discrimination “in lower levels of the labour market” means that parents encourage children to pursue higher education (Salikutluk, 2016, p. 583). A fourth, also structural, explanation is the *information deficit* explanation (Kao & Tienda, 1998), which proposes that a lack of information and knowledge about the education system in the country of settlement “can bias the perception of students’ chances in schools and thus explain why minorities have unrealistically optimistic or pessimistic aspirations” (Salikutluk, 2016, p. 583). A fifth explanation underlines that “*ethnic capital*” can compensate for disadvantage and support educational goals (Khattab, 2015, p. 734, see also Naidoo, 2015).

Finally, explanations emphasize *the role of significant others*, especially parents, in high educational aspirations (Friberg, 2016). The role of significant others is not necessarily a separate explanation, since they can be operative in the other explanations (e.g., the intergenerational “project” for social mobility). Broadly speaking, aspirations generally do not emerge in a vacuum, but are “influenced by significant others” either through direct communication or as role models (Kao & Tienda, 1998, p. 352), just as hope is not a sole individual characteristic, but also constructed and nourished through cultural, religious, and social mediators (cf. Eggerman & Panter-Brick, 2010; Panter-Brick & Eggerman, 2012).

### Studies on young refugees’ educational and vocational aspirations

Whereas most research on immigrant/descendant youth and education does not focus specifically on refugees, research on refugee education has grown in recent years, and also includes studies on educational and vocational aspirations. Several of these studies suggest that additional explanations to those outlined above may (also) contribute to refugees’ often high aspirations.^6^ Some Norwegian studies found that unaccompanied minors’ vocational aspirations can be influenced by aims of economic independence, of supporting family in dire situations abroad (Bovollen, 2016; Oppedal et al., 2017) and/or of having necessary income for family reunification (Bovollen, 2016). Studies from the US and Canada (McWilliams & Bonet, 2016; Shakya et al., 2010) have found that aspirations can be influenced by a desire to economically support family members who struggle in the new country (e.g., due to health, language and economy).

Furthermore, several studies find that educational aspirations can be motivated by altruism—wanting to benefit others in the country of origin (McWilliams & Bonet, 2016; Oppedal et al., 2017; Tlhabano & Schweitzer, 2007) or striving with the aim of a well-paid job that can enable them to contribute to others in the new country (Shakya et al., 2010).

Studies on refugees have, moreover, highlighted temporal aspects of aspirations. Some studies highlight the importance of educational aspirations also before the migration, finding as a pattern that the main reason for the flight is seeking safety, followed by wanting an education that is not possible in war-torn/politically unstable countries (Tlhabano & Schweitzer, 2007; Vervliet et al., 2015). Other studies show how aspirations may increase or diminish in the new country, facilitated, respectively, by positive school experiences and barriers to education (McWilliams & Bonet, 2016; Shakya et al., 2010).

Some of the other identified potential influences on young refugees’ vocational aspirations are current mastery at school (Bovollen, 2016), language challenges (Tlhabano & Schweitzer, 2007) and educational expectations by family members (due to income and/or status) (Bovollen, 2016). One study found that vocational and educational aspirations were more related to “gender, age, or familial position than to//‘refugee’ status” (Vervliet et al., 2015, p. 341).^7^

In several of these studies among refugees, the dual frame of reference explanation seems especially relevant. In some explanations, significant others are also important. Meanwhile, the findings on striving for social mobility in these studies seem influenced by the refugee experience and thus diverge from explanations of labour migrants’ intergenerational mobility project depicted in the immigrant optimism theory. Instead, an aim for social mobility in these refugee studies relates to aims for personal economic security or economically supporting others abroad or in the new country.

## Design and methods

### The study

The discussed findings derive from the qualitative research project Transitions Upon Resettlement in Norway (TURIN), which is a sub-study of the Nordic research project Coming of Age in Exile (CAGE).

The TURIN study’s primary objective is to gain insight into what may promote or inhibit educational and psychosocial transitions among young refugees in the resettlement phase. The study was carried out by researchers at the Norwegian Centre for Violence and Traumatic Stress Studies (NKVTS) and the University of South-Eastern Norway (USN). This article is based on data collected by the NKVTS team at three upper secondary schools in the Greater Oslo Region.

### Method

The study adopted a qualitative, ethnographically oriented, study design, based on interviews and participant observation in the participating schools. Two researchers, including Pastoor, and a research assistant conducted semi-structured interviews (lasting approximately 45 min to 2 h) with refugee students and school staff at one school each.

The main interview guide themes for young refugees included questions on everyday life, school experiences, psychosocial aspects of resettlement, life before arrival in Norway and future plans and aspirations.

### Data collection and participants

#### Access and recruitment

Based on the acquired consent from the Norwegian Data Inspectorate, the county education authorities were contacted enquiring about whether they were interested in participating in the study, and which schools would be relevant to approach.^8^ All the schools contacted agreed to participate, welcomed the researchers into the classrooms, and facilitated interviewee recruitments. The interviewers did not approach the refugee students directly. Teachers first introduced the researcher to the class, and the researcher then presented the study to the students. Frequent school visits enabled teachers, students, and researchers to become acquainted. Students whom the researchers identified as refugees were then invited to participate in the study. This “stepwise” recruitment was chosen in order to develop reciprocal trust, which was especially important since the interviews could entail sharing sensitive or personal information. The researchers had informal conversations with some of the interviewees prior to and after the interviews. Trust is important in all research, but in research on refugees building and maintaining trust is especially important throughout the research process since the refugee experience can lead to a strong distrust (Eide et al., 2018; Hynes, 2003).

#### Participants

In the three Greater Oslo region schools, 26 individual interviews were conducted with 26 refugees with 6 years or less residency in Norway. The interviewees were between 16 and 25 years old (most were under 18 upon arrival). The sample includes nine unaccompanied minors, eight individuals family reunified with refugees, four former asylum seekers arriving without family, two former asylum seekers arriving with family, and two UN resettled refugees. The interviewees came from Eritrea (9), Somalia (7), Syria (4), Afghanistan (3), and three other countries.^9^ The students’ prior education varied greatly, encompassing no/no fulltime formal primary school education (9), completed primary school (3), completion/almost completion of the equivalent of Norwegian lower secondary school (9) to having had completed/almost completed upper secondary school (4). Around one-third lived with their families. Most of the other interviewees lived in supported residential settings or in more informal shared accommodation with flatmates. One interviewee lived alone.

In the three schools, interviews were also conducted with 30 teachers and other school staff. We refer briefly to some relevant parts of this dataset, and otherwise use it as contextual background material.

Each participant signed a consent form (approved by the Norwegian Data Inspectorate), after having been informed about the study’s aim, data collection, data management, and their right to withdraw from the study at any time.

#### Data handling and analysis

The article’s findings are primarily based on analysis of interview transcripts. Professional transcribers transcribed the interviews verbatim. All interviews were coded in NVivo, a software program for qualitative and mixed-methods data. The program supports the researcher in organizing and analysing qualitative findings, for example, during the process of moving from the researcher’s initial, descriptive themes towards more abstract, analytic categories. Most of the coding was conducted by Lynnebakke, in close, regular dialogue with Pastoor about the emerging analysis. For closer access to nonverbal cues such as tone of voice and length of hesitations, most of the coding was conducted whilst listening to the recorded interviews. This seemed to reduce the distance to interviewees by obtaining more information about their personalities and aspects of the interview situations, such as the degree of rapport.

The coding began with open codes, followed by merging codes in overarching categories and reorganizing the codes into coding trees through several cycles (Saldaña, 2016). In addition, each interview was summarized in a Word document. After coding, central interviews for the article’s topic were reread in their entirety. Theoretical and empirical memos were written and discussed throughout the coding process. The analytical approach used during coding aligns with Timmermans & Tavory’s (2012) approach of combining abductive reasoning with grounded theory (GT) methods such as meticulous coding and the use of memos. Abduction involves the active use of theories with which the researcher is cognizant of to analyse unexpected findings, whilst GT methods foster empirical closeness and defamiliarising oneself from habitual everyday thinking and favourite theories (Timmermans & Tavory, 2012).

Notwithstanding our initial resilience lens, the extent to which many young interviewees expressed self-efficacy at school was striking. The “educational resilience” concept thus became a valuable concept brought in at a later stage of analysis, as did theories on immigrant youth’s educational aspirations.

The student accounts on motivational reasons outlined in the findings section (third sub-heading) were developed through a process of analytic induction (Katz, 2001), entailing that all interviewee statements in our data can be grouped under these categories. After outlining these reasons, we describe two in-depth interviewee examples in order to provide more coherent individual accounts that show firstly, how there can be a confluence of several motivational reasons, and secondly, how past and present experiences can influence educational aspirations. The in-depth examples have been purposefully selected to highlight how “the refugee experience” can influence aspirations. The examples illustrate diversity within the large refugee category and are not representative exemplars of our whole data set. Whilst these interviewees share some features, experiences and views with some of the other interviewees, these accounts of two unique individual trajectories point to theoretical gaps regarding the aspirations of refugee youth that cannot be fully explained by the current theories about the educational aspirations of immigrant youth.

All quotes used in this article are translated from Norwegian. For anonymisation purposes, we have omitted certain details (e.g., from the flight) from the accounts.

### Limitations, challenges and ethics

The interviews varied in length and detail. In some of the interviews, interviewees’ Norwegian language levels were a challenge. This resulted in shorter, often affirmative answers that could not be used in the analysis. The analysis draws primarily on interviews with rich, more elaborate accounts on the article topics in order to report interviewees’ experiences as much as possible 0n their own terms.

, The relatively formal interview setting in schools may also have influenced some students’ degree of openness. Additionally, some young refugees may not want to disclose personal reflections and experiences due to both common adolescence development processes and healing processes. Strategies for dealing with psychological distress and trauma can vary over time, where talking about distress can seem helpful to healing at some times and disruptive at other times (Kohli, 2006). Moreover, individuals vary in their coping strategies and how they experience to talk about personal, difficult topics. Finally, it could be that politeness, interviewee/interviewer age differences and/or trust and rapport issues influenced shorter comments in some of the interviews (cf. Wernesjö, 2012).

Trust often increases over time, and some research has found that young refugees may give surface narrative of themselves in the beginning (Hynes, 2003). In this perspective, a limitation with the study is that the young refugees were only interviewed once. On the other hand, the researchers were present at the schools for some time before conducting the interviews and had informal conversations with some interviewees prior to the interviews.

Some refugees may want to have a self-presentation that conveys resilience and strength as a direct counter-reaction to that that refugees are often generally are conceived through gaze of pity, vulnerability, or charity (cf. Chase et al., 2020; Hynes, 2003; Wernesjö, 2020). Furthermore, some individuals can deem it as stigmatizing to be called a refugee, or may not wish to not be identified as a refugee because they want to “move on from their past traumas” (Stevenson & Willott, 2007, p. 684). Researchers’ focus on the refugee experience of individuals comes with a risk of reductionism and essentialising (Chase et al., 2020). It is important that research emphasizes not only many young refugees’ extraordinary experiences but also acknowledge that they are “ordinary people driven by ordinary desires” (Robinson & Segrott, 2002, p. 64, as cited in Kohli, 2006, p. 708). Since this study concerns transitions into a new country, it included questions on how participants experienced to be a newcomer and on pre-migration experiences. However, most of the interview guide focused on current everyday experiences (e.g., school experiences, living situation). Questions on pre-migration experiences were usually asked at the end of the interview unless interviewees raised and elaborated on these topics earlier. Pre-migration questions focused mainly on ordinary experiences—addressing, e.g., family background, education, and work. Students were usually asked about their migration route and the locality of the asylum reception centre they had stayed at in Norway (if applicable) in order to gain knowledge on where they had lived/stayed and on previous education provisions. Beyond this, the intention was that interviewees would only elaborate on their experiences prior to resettlement if they wanted to.

In our empirical presentation of students’ accounts of motivation reasons, we aim to strike a balance between on the one hand, discussing when the refugee experience seems to impact on aspirations, and on the other hand to underscore the ordinariness of many of the other motivation reasons.

The TURIN study’s focus on psychosocial transitions included the topic of mental health. The researchers aimed at addressing mental health in a sensitive way by asking general questions on daily functioning and asking follow-up questions if relevant. The follow-up questions were important, since e.g., poor concentration not necessarily was related to the refugee experience but due to more common, ordinary experiences.

## Findings: analysis and discussion

### Facilitators of high aspirations

As stated, we conceive educational resilience as promoted not only by individual traits but also by relational and environmental factors. As we now outline, students’ challenges often coexisted with positive expectations of their educational mastery. Below, we also present supportive factors in the interviewees’ lives that seemed to contribute to their educational resilience.

Many students described being very focused on school, having high educational aspirations and/or working hard to achieve these aspirations. Interviewees expressed such notions regardless of the educational level they aspired to. Very few described feeling overwhelmed by school tasks. They especially described challenges related to learning (in) a new language but tended to describe these challenges as manageable. Few interviewees reported psychological distress at the time of the interview to the extent of this affecting their schoolwork negatively, such as problems with concentration and/or sleep. However, several reported having experienced strong psychological distress at an earlier stage in Norway, for example, before being granted a residence permit. Some stated that their mental health had improved due to previous help from health professionals.

Researchers of unaccompanied minors underscore that vulnerability can coexist with resilience, agency, and resourcefulness (Çelikaksoy & Wadensjö, 2019; Chase et al., 2020; Eide & Hjern, 2013; Watters, 2008). In our study, many of the same interviewees who reported high aspirations also shared dramatic flight experiences, told about loss of people left behind and/or that they wanted more social contacts with majority Norwegians. In addition, the classroom observations suggested that some refugee students experienced repeated difficulties in keeping up with the class.

Several students stated that an individual’s hard work is what determines educational outcomes. Such statements indicated self-efficacy, that is, beliefs about an individual’s “capabilities to produce designated levels of performance that exercise influence over events that affect [one’s] lives” (Bandura, 1994). For many interviewees, one likely source of self-efficacy was previous mastery experiences (Bandura, 1994)—as several had been good at school in their country of origin. In addition, previous educational levels were a resource for many interviewees. Several of those with lower secondary or upper secondary school from the country of origin reported that they experienced continuity between previous schooling and current educational demands and reported that the Norwegian language was the main challenge. Furthermore, supportive others inside and outside school seemed to contribute to many interviewees’ expressed educational resilience and self-efficacy, in line with self-efficacy theory (Bandura, 1994). Several interviewees shared how individual family members believed in and supported their educational aspirations without putting what seemed unachievable pressure on them. Usually, these family members lived in Norway, but some told about support from family members abroad. Interviewees frequently reported what appeared to be intrinsic motivational reasons. In these reports, parents seemed to play a role as motivators and supporters of their aspirations, but left the decision about which kind of education up to the youth. It seemed to be the search for a good, stable life rather than social mobility and status per se which was most central for interviewees (at least explicitly reported), and in this quest they experienced family support.^10^ These findings contrasted some previous Norwegian studies, which have found that the high aspirations of migrant/descendant youth can be influenced by their parents’ ambitions and/or sacrifices through mechanisms of feeling a sense of indebtedness (Fekjær & Leirvik, 2011; Leirvik, 2010), wanting to make parents proud (Friberg, 2016) and/or wanting to live up to parents’ high expectations (Fangen, 2010; Finne, 2010). Only one interviewee reported feeling indebted to her parents. Notably, this interviewee also diverged from the broader empirical patterns in that she for various reasons (including parents’ educational pressure) seemed most overwhelmed with school and other aspects of life.

Several interviewees who reported high educational aspirations and a sense of mastery also reported having a strong religious faith. Religion has been identified as among numerous factors that can promote resilience (Hutchinson & Dorsett, 2012; Sleijpen et al., 2016). This may relate to religion as a source of personal strength (e.g., accepting adversity and experiencing continuity) (Sleijpen et al., 2015) and/or as social networks and support (cf. Betancourt & Khan, 2008; Hirschman, 2004; Wilkinson et al., 2017). Whereas some interviewees participated in religious communities, those who explicitly shared that their aspirations were aided by religion referred to individual faith-based aspects rather than social aspects. Some stated that they did their best, but also had faith in a higher power to decide the best future outcomes in their lives.

### Motivation and time

Our findings demonstrated that high motivational levels and (educational) resilience are not static individual traits. These findings, again, pointed to the importance of a supportive environment for educational aspirations. Moreover, the findings showed how motivation can vary over time due to the refugee experience. Some interviewees with current high aspirations told about how they previously had been rather indifferent about school in the asylum period, when their future was more unknown. Moreover, teachers reported that it was sometimes difficult to support sustained motivation of recently arrived immigrants, including refugees. Some commented that they had seen many recently arrived students come with high optimism that later decreased when school was more difficult than expected. Several teachers emphasized the interrelation between mastery experiences and motivation, and some commented on how educational achievements can influence mental health and vice versa, as also found in much research (see Mælan et al., 2018). One preparatory class teacher remarked:

(Int.: Have you found that the physical and/or mental health of the [refugee] students has negatively affected their school functioning?) [firmly] Yes … both.//Most refugees I have taught have struggled psychologically. Often, it’s a bit like they’re in an early phase of being in love when they come to Norway, because now they have arrived in a safe country and everything is going to be so good and [they can have] very high expectations and suchlike. And then they learn quite a lot of Norwegian in the beginning, but then they see that things are more complicated [than they expected] and that they need to learn much more. And it wasn’t quite the way they had thought, and then they get a bit burnt out, eh, and it’s like such normal migration problems, and those who in addition have traumas get hit especially hard. Then they can be incapacitated for learning for shorter or longer time periods.

The statement highlights learning/motivation processes that relate to both the migration experience (shared by all immigrant youth) and the refugee experience—highlighting how previous trauma may make it even more demanding to sustain motivation over time.

### Student accounts on reasons for motivation

Young refugees gave diverse reasons for their high educational aspirations. Interviewees were usually motivated by several reasons, but often emphasized one reason. Some reasons may be shared by all youth, whereas other reasons may be shared with other migrant categories. Finally, some reasons reflect the refugee experience specifically. The latter suggests that a quest for understanding migrant youth’s often high aspirations should include consideration of the relevance of migration history.

***1. Motivated by the aim of an economically secure and stable future***

The most commonly stated motivations among student interviewees involved aims for a stable, economically secure future. This included statements about aiming for a specific job (due to personal strengths/interests and/or good job opportunities); being independent; “being someone” and not working in vulnerable lower-skilled jobs.

Such aims are shared by many young people, regardless of background. Some interviewees linked their aims for future stability to what they described as Norwegian or universal norms of the importance of working hard to reach one’s goals. On the other hand, several interviewees reported not taking education for granted, which could relate to the refugee experience. We will return to this.

***2. Motivated by social relations/significant others***

This category included statements about being motivated by aims of providing economically for one’s family (in Norway or abroad); being able to pay for family members’ visit to Norway; the encouragement and praise from one’s teacher; being a role model for siblings and being accepted as a potential son-in-law. A few of these explanations relate to the migration experience more broadly.

***3. Motivated by wanting to contribute to society***

This motivation category included statements on:

- *wanting to make a difference in Norway or other countries*
- *using one’s personal experience to the benefit of others*
- *being part of Norwegian society*

The last bullet point is worth highlighting, since, to our knowledge, it is usually not addressed in migration-related explanations for high aspirations. Tahir is 25-year old and came to Norway as an asylum seeker. He explained:

(Int.: Do you feel at home here?) Home? (Int.: Yes?) One has to accept reality. If I say home … Yes, no, well. No, because of the issue with Mahad [media case], have you seen the case? [He is] from Somalia [and after] 17 years [in Norway] suddenly he loses his right to residence.//No, [I don't feel at home] if I think that way. But I am content. I feel like I am part of society because I go to school, I am getting an education, and I work. I pay tax. So I feel a bit positive. (Int.: Contributing so society like others, mm.) Yes,//I’m young, so I have opportunities and can work, right, instead of being on benefits and saying, ‘I don’t have this, I don’t have that’. I feel a bit, like, positive.

Tahir was the only interviewee who explicitly stated that he expected labour market discrimination. A broader reading of Tahir’s interview indicated that the quote had an undercurrent of claiming belonging by proving himself a decent citizen (see also Wernesjö, 2020). Expectations of discrimination seemed to play a different role for Tahir than in the blocked opportunities theory, however, where such expectations fuel hard work to pursue higher education (Salikutluk, 2016). Tahir instead framed the need to work hard as a universal rule.

***4. Motivated by personal development***

This category included statements of motivation due to, for example:

- *a joy of learning*
- *aiming for a certain education because of strengths in certain school subjects*
- *learning from one’s mistakes*
- Again, we highlight Tahir’s interview, to show how different reasons may interweave and jointly create high educational aspirations. Whilst Tahir referred to labour market discrimination, he seemed more focused on the fact that education can provide opportunities for personal development:

Since I came to Norway, I have developed every year, right. I feel kind of, a bit better because one has opportunities, right. One has the same rights, one perhaps doesn’t have 100 per cent the same rights in reality as a Norwegian but one has the opportunity to develop oneself, right.

This statement also shows how gratitude and cherishing opportunities for personal development can involve implicit comparison—i.e., a dual frame of reference—with conditions in the country of origin, leading us to the final motivational category.

***5. Motivated by previous experiences and conditions in the country of origin***

Statements in this category included comments on:

- *Different gender roles compared to the country of origin*, underlining educational and occupational opportunities for girls/women in Norway
- *Humanitarianism due to flight experiences and wanting to make a change in war-torn countries*. This overlaps with previous research findings on pre-migration educational aspirations (McWilliams & Bonet, 2016).
- *Comparing educational opportunities in Norway with (a lack of) opportunities in the country of origin*. Several interviewees reported that school was one of the best things about Norway and some reported appreciation for that school and most higher education is free in Norway. These findings align with the findings of Shakya et al. (2010) from Canada. Furthermore, several of these reasons align with the dual frame of reference theory but highlight aspects other than socioeconomic comparisons. This is seen in a statement by the 19-year-old preparatory class student Tefsaye:
  In Norway, there’s a lot of freedom. You can go to school. For example,//where I come from, you can’t go to school.//Right, Norway is a democratic country, you can learn anything. I am free. (Int.: But in your Eritrea you couldn’t go to school?) You can go to school, but//whether you go to school or not, you don’t get … Anyway, it will be the military, right.//You don’t have freedom, actually.
- *Wanting to give back to the country of residence* (see Salam’s story, below)
- *Making migration worthwhile*. Yonas, an unaccompanied refugee minor from Eritrea, wanted to make an extra effort to improve himself and to build something new because of his exile:
  Many have lived here for 10–15 years and don’t do anything. They just exist//. They haven’t done anything, they haven’t bought an apartment, they haven’t developed—they just live and drink and eat in town.//If I live with my family in Eritrea, that is not a problem, but when one moves and loses one’s family—I must think of myself—why I moved from my family. I must think.

Yonas’ statement seems to tangent the findings of McWilliams and Bonet (2016), where some refugees saw it as a moral obligation to use one’s educational opportunities. The quote could also be framed as a way of creating coherence and meaning in one's life (Antonovsky, 1987).

### Narratives of past and present influences on educational aspirations

The following two narratives aim to show how key events and reasons can jointly shape educational aspirations in unique individual trajectories. During the presentation, we consider the relevance of the refugee experience for these interviewees’ aspirations.

#### Lilah’s story

Lilah is a twenty-year-old female who attends preparatory class. She came to Norway with her father as an asylum seeker from Syria two years ago. Later, her mother, siblings, and some other family members were reunited in Norway. Lilah lives with her parents and siblings and enjoys spending time with her family.

School and education mean a lot to Lilah. Her narrative exemplifies several motivational reasons for high educational aspirations. First, Lilah says that what she likes best about Norway are her educational opportunities and the *gender roles*. She explains that in Syria, “girls must stay at home, cook for the family and look after smaller siblings” whilst in Norway, girls “always have to go to school and then work.” This highlights the dual frame of reference. Second, Lilah states that it is good to attend school because if family members in Syria call and ask her parents about her, they will be impressed and “happy when they hear that I study, keep working”, since a lot of people in Syria are not able to do this because of the war. On the surface, this could be read as an example of migration as a family project for social mobility, and points in a similar direction to some other research (Friberg, 2016), in that Lilah wants to make her parents, or perhaps her relatives in Syria, proud. However, another layer of the statement suggests that Lilah is motivated by wanting to make the most of opportunities she would not have in the war-torn country of origin: making the flight worthwhile. The statement suggests that her flight experience is accompanied by a sense of responsibility and/or gratitude (or even guilt for being safe when relatives are not (Labby, 2016)), which motivates her to not squander her new opportunities.

Third, Lilah is motivated by *personal development*, stating that what is best with school is not only learning subjects, but also that she can learn from her mistakes. Fourth, Lilah states that she wants to attend upper secondary school because that is common in Norway. Finally, she is motivated by aims of having *economic security* and freedom, explaining that she wants to “get a job, earn money, buy an apartment, have a large family” and being able to afford to visit family abroad. Her high aspirations thus reflect both the refugee experience and many youth’s aims of economic independence.

#### Salam’s story

Salam is a 23-year-old male from a Middle Eastern country who came to Norway as an asylum seeker four years ago. After leaving his war-torn country of origin, he worked and lived in two other countries. Salam’s flight was long and turbulent; however, he also had several experiences of being helped out of difficult and dramatic situations during the flight.

Salam had no schooling until he was a teenager. However, he is good at learning new languages, which seems to have helped him in Norway. Except for one subject, he reports not finding school especially difficult. When he was waiting to be granted asylum/residency, he had concentration problems, but this did not trouble him later. Despite lack of prior schooling—or perhaps because of it—Salam was focused on getting an education even before he came to Norway. His narrative of the flight through different European countries reads as an account where his agency for education and a safe, stable future intermingled with coincidences and information he received during the flight. For example, when he told someone that he wanted to get an education, he was advised to seek asylum in another Nordic country, but upon arrival there, he had a chance meeting with another asylum seeker from his country of origin who said Norway “is better”. Together with other coincidences, this led Salam to move to Norway. The morning after his arrival, someone knocked on his door in the transit centre and told him he was going to school. Salam had not expected school so soon but was eager to go. He explains that he has worked hard since he was a child, often under harsh conditions, and that this motivates him to create a different future for himself:

I have never said no to school,//I would never have been like ‘no, now I’m not going to school.’ When they said “you have an offer”, I said “ok, let’s go to school”. Because I have worked too much under people, for example, as a slave. I have always worked as a slave in other countries.//When you understand something, you are the boss, right. When you don’t understand anything, you just have to work. Right. So I always thought about becoming a boss one day, right.//So I started going to school.

Salam’s statement reflects a dual frame of reference where socioeconomic comparisons are central, thus potentially being a sentiment shared by individuals of other migrant categories. Other parts of his account reflect the refugee experience. Salam has worked a great deal in addition to attending school, and been able to buy a car, and has plans to buy an apartment with his wife. He aspires to a higher education profession within health care, motivated by a strong gratitude and desire to give back to Norway.

Imagine, seven years ago when I came to Norway, I had nothing! I wore clothes I maybe hadn’t washed in a month, right. I slept on the street and suchlike.//Then [it] began. And today I have a car, my house is filled with all I need. Clothes, money, school, education. Health.//Maybe I couldn’t have achieved as much in other countries as in Norway. So, in my thoughts, Norway was the country I needed. And in the future, I [will] try to do my best for this country also, because if I’d stayed in [country of origin] today, I would have been killed, or if I hadn’t been killed, I’d have no education//. In other countries [he has transited through] also.//I will try to complete an education and help and work back for this country, right.

This statement has a strong ring of relief. Whilst Salam has aimed to make a safer and better future for himself, his reasons for high aspirations also changed after migration to also include a sense of gratitude.

## Concluding discussion and reflections

During adolescence, young people must make fundamental decisions about their future, such as further education and career (Rutter, 2003). The predicament of “late arrival” adolescent refugees is that they must accomplish much in a short time. They must learn a new language, catch up in the country’s education system and, eventually, choose the appropriate school programme with respect to post-secondary opportunities. Educational outcomes should not just be left to the responsibility of student motivation; educational policies and structural factors are crucial. Nevertheless, high motivation and high aspirations can be strong resources when encountering educational challenges in a new country.

This article has explored the question *what shapes young refugees’ often high educational aspirations?* We have accounted for a broad set of potential reasons in our own research and other studies. Our findings showed reasons that reflect the ordinary desires of many young people; reasons that may be shared with other migrant categories and reasons that highlight the refugee experience.

In the introduction, we highlighted that school and education potentially have additional meanings for young refugees because of the demanding refugee experience and the stabilizing role school can play. The findings on the potential relevance of the refugee experience for aspirations suggest gaps in current theories on immigrant youth’s often high educational aspirations. We saw that the refugee experience may influence aspirations and the meaning of education in several ways such as e.g., being motivated to make the flight worthwhile or due to altruism that is fuelled by conditions in the country of origin, as also found in other research on refugees. We also presented how Salam’s gratitude for safety and social mobility opportunities in Norway motivated him to give back. Furthermore, we saw that for some young refugees, being motivated by personal development can be strengthened by a dual frame of reference and a strong sense of gratitude for a “new life”. This also exemplified that individuals can have several motivation reasons and that different reasons can interact.

Some of the findings can be conceived as “immigrant optimism”, but the intergenerational project for social mobility was not central in the findings.

The contrast between our findings and other Norwegian studies on the role of parents in educational aspirations may partly reflect the different country backgrounds of the research participants in the studies, as educational aims (e.g., aiming for high-status jobs/educations) can vary greatly among different country backgrounds (e.g., Østberg, 2010; Teney et al., 2013). This may also partly explain why, in our findings, *ethnic explanations* were not prominent reasons for high aspirations.

In numerous places, we have commented on ways the findings support the dual frame of reference theory. Some of these comparisons highlighted coming from war-torn/unstable settings. The findings suggest that discussions on “immigrant optimism” should take into account temporal aspects of aspirations. Optimism is often not a constant of the refugee experience. Moreover, the findings showed that a dual frame of reference may involve not only comparisons with prior experiences and conditions in the country of origin, but also with different stages of the refugee experience, such as the flight and asylum-seeking process. This is demonstrated by reports on becoming more motivated for school after being granted residency. Furthermore, both Salam’s account of gratitude and the findings from previous studies (McWilliams & Bonet, 2016; Shakya et al., 2010) suggest that resettlement experiences and structural factors in the new country can reduce or increase aspirations. Teacher statements also pointed to changes in aspirations and motivation levels during different stages of resettlement. These statements indicate that some students’ optimism might partly reflect a life stage marked by increased stability and being at an early stage of resettlement. To sustain motivation and contribute to educational outcomes, it is important with educational policies that aim to level out inequities (cf. Huddleston & Wolffhardt, 2016, p. 31) and supportive others inside and outside school, aligning with the role of environmentally protective factors for educational resilience (Bahram et al., 2014).

Research into the often high educational aspirations of immigrant youth pays due attention to differences within the “immigrant population”, especially socioeconomic and country backgrounds (e.g., Bakken & Hyggen, 2018). An implication of our findings and the other outlined studies on refugees’ educational and vocational aspirations is that *migration reason* should also be theoretically explored and empirically controlled for in surveys, and further explored in qualitative research.

Moreover, the findings of this and other studies on young refugees bring attention to temporal aspects of the refugee experience and how this may influence aspirations. Whilst research has addressed generational changes in educational aspirations over time (cf. Suarez-Orozco et al., 2009), longitudinal studies should address changes in individual aspiration over time, and how these changes may relate to different stages of the refugee experience.

## Notes

1. We refer to all interviewees by pseudonyms.

2. We acknowledge the large heterogeneity of experiences and sub-categories within the refugee category.

3. For more information, see Tørslev and Børsch (2017).

4. There are large country background differences on a group-level that cannot be fully be explained by controlling for educational backgrounds and language levels (Østberg, 2010).

5. Concerning socioeconomic comparisons, this explanation is most applicable for migrants whom previously had lower socioeconomic positions in the country of origin, as becomes evident in Kindt (2017).

6. We focus here on educational and vocational aspiration studies from the upper secondary/high school level, and on studies that include or focus solely on the resettlement context.

7. Transcription conventions://indicates that some text has been left out of the excerpt, [text] indicates additional information. Ellipses are used to indicate a hesitation or pause.

8. In Norway, the county authorities are responsible for providing upper secondary education.

9. Not specified for anonymity reasons.

10. In interviews marked by shorter answers, it is possible that indebtedness and/or high family pressure was not reported in an interview with a relatively unknown person.
